# Occurrence of multidrug-resistant bacteria and clinically important β-lactamase resistance genes in giant freshwater prawn (<em>Macrobrachium rosenbergii</em>) aquaculture ponds in Thailand

**DOI:** 10.14202/vetworld.2026.2722-2733

**Published:** 2026-07-06

**Authors:** Keeravit Petjul, Prasit Khunsanit, Tanaphoom Boonmee, Anupong Tankrathok, Urai Koollboon, Nattapon Kan-a-roon

**Affiliations:** 1 Department of Interdisciplinary Agriculture, Faculty of Agricultural Technology, Kalasin University, Mueang District, Kalasin Province 46000, Thailand.; 2 Department of Biotechnology, Faculty of Agricultural Technology, Kalasin University, Mueang District, Kalasin Province 46000, Thailand.; 3 Department of Fisheries Technology, Faculty of Agricultural Technology, Kalasin University, Mueang District, Kalasin Province 46000, Thailand.

**Keywords:** Aeromonas veronii, antimicrobial resistance, aquaculture, β-lactamase genes, giant freshwater prawn, multidrug-resistant bacteria, One Health, Thailand

## Abstract

**Background and Aim:**

The rapid expansion of giant freshwater prawn (*Macrobrachium rosenbergii*) aquaculture has raised concerns regarding the emergence and dissemination of antimicrobial resistance (AMR) in aquatic ecosystems. However, information regarding antibiotic-resistant bacteria and resistance genes in freshwater prawn earthen pond systems in Northeastern Thailand remains limited. This study aimed to isolate and characterize antibiotic-resistant bacteria from giant freshwater prawn aquaculture ponds in Kalasin Province, Thailand, and to determine the occurrence of clinically important β-lactamase resistance genes.

**Materials and Methods:**

Water samples were collected quarterly from nine earthen ponds located in three districts of Kalasin Province, Thailand, between July 2024 and June 2025. Bacterial isolates were recovered using ampicillin-supplemented selective media and identified through *16S rRNA* gene sequencing. Antimicrobial susceptibility was evaluated using agar disk diffusion according to Clinical and Laboratory Standards Institute guidelines. Polymerase chain reaction assays were performed to detect β-lactamase genes, including *blaTEM*, *blaSHV*, *blaOXA*, *blaKPC-2*, *blaNDM-1*, and *blaIMP*.

**Results:**

Twenty antibiotic-resistant isolates representing eight bacterial species belonging to five genera were identified. *Aeromonas veronii* was the predominant species, accounting for six isolates. Fourteen isolates (70.0%; 95% confidence interval: 45.7–88.1%) exhibited multidrug resistance to at least three antimicrobial classes. Resistance was particularly common against ampicillin, vancomycin, and rifampicin. Molecular analysis revealed the presence of clinically important β-lactamase genes, mainly *blaSHV* and *blaKPC-2*. Several isolates carried these genes, and *A. veronii* isolate MSS1 co-harbored *blaSHV* and *blaKPC-2*, indicating the possible clustering of resistance determinants. To the best of our knowledge, this study represents the first report of *blaKPC-2*-positive bacteria isolated from *M. rosenbergii* aquaculture ponds in Thailand.

**Conclusion:**

The detection of multidrug-resistant bacteria and clinically relevant β-lactamase genes highlights the role of freshwater prawn aquaculture systems as environmental reservoirs of AMR. These findings provide baseline information for AMR surveillance in Thailand and emphasize the need for improved antimicrobial stewardship, enhanced biosecurity measures, and sustainable disease management strategies within a One Health framework. Further investigations employing metagenomics and whole-genome sequencing are warranted to elucidate resistance dissemination mechanisms.

## INTRODUCTION

Aquaculture has become a cornerstone of global food production, with freshwater prawn farming representing a rapidly growing sector in Southeast Asia. The giant freshwater prawn (*Macrobrachium rosenbergii*) is one of the most commercially important aquaculture species because of its rapid growth, high market value, and adaptability to diverse farming systems [1, 2]. In Northeastern Thailand, particularly in Kalasin Province, freshwater prawn farming contributes substantially to rural livelihoods and local food security. However, the intensification of aquaculture production has led to increasing challenges associated with disease outbreaks and environmental health. Opportunistic bacterial pathogens, particularly *Aeromonas* spp., are frequently associated with motile aeromonad septicemia and other infectious diseases in cultured prawns [3, 4]. Consequently, antibiotics are commonly used for disease prevention and treatment in aquaculture systems, often without strict veterinary oversight, raising growing concerns about antimicrobial resistance (AMR), antibiotic-resistant bacteria (ARB), and antibiotic resistance genes (ARGs) in aquatic environments [5, 6]. Environmental dissemination of clinically important resistance genes, including *blaSHV, blaOXA,* and *blaKPC-2*, has increasingly been reported in aquatic bacterial species such as *Aeromonas* spp., *Klebsiella* spp., and *Enterobacter* spp., highlighting the potential public health risks associated with aquaculture-associated AMR [7–10]. In response to these concerns, the World Health Organization has emphasized the importance of integrated AMR surveillance under a One Health framework [11].

Despite growing global concern about AMR in aquaculture, most investigations in Thailand have focused primarily on marine shrimp farming systems or cage-cultured fish, including studies associated with Lam Pao Dam and coastal aquaculture regions. In contrast, earthen pond systems used for culturing *M. rosenbergii* in Northeastern Thailand remain poorly characterized with respect to profiles and the occurrence of clinically relevant ARGs. This knowledge gap is particularly important because small-scale freshwater prawn farming represents a major source of income and food security for rural communities in Kalasin Province and surrounding regions. Furthermore, previous studies have generally investigated bacterial diversity or antimicrobial susceptibility independently, whereas only a limited number have integrated culture-based bacterial isolation, molecular identification using *16S rRNA* gene sequencing, antimicrobial susceptibility profiling, and targeted detection of clinically important β-lactamase genes in freshwater prawn aquaculture systems. Information regarding carbapenemase-associated genes, particularly *blaKPC-2*, in environmental isolates from freshwater prawn ponds remains scarce in Thailand. In addition, the occurrence and distribution of clinically important β-lactamase genes among ARB associated with freshwater prawn farming systems have not been comprehensively characterized. This lack of information limits ecological risk assessment and hinders the development of effective surveillance and antimicrobial stewardship strategies under a One Health framework.

Therefore, this study aimed to isolate and identify ARB from *M. rosenbergii* aquaculture ponds in Kalasin Province, Thailand, and to characterize their antimicrobial susceptibility patterns and associated β-lactamase resistance genes, including *blaSHV* and *blaKPC-2*. By integrating culture-based bacterial isolation, molecular identification using *16S rRNA* gene sequencing, antimicrobial susceptibility testing, and targeted polymerase chain reaction (PCR)-based detection of resistance genes, this study provides important baseline information regarding multidrug-resistant (MDR) bacteria and clinically relevant ARGs in freshwater aquaculture environments. The findings are expected to improve the understanding of AMR dissemination in small-scale freshwater aquaculture systems and support future surveillance and antimicrobial stewardship strategies in Thailand under a One Health perspective.

## MATERIALS AND METHODS

### Ethical approval

This study was conducted in accordance with the ethical principles and research guidelines established by Kalasin University, Thailand. The study protocol was reviewed and approved by the Research and Development Institute Committee of Kalasin University. All procedures complied with applicable national regulations and institutional standards for microbiological and environmental research. Laboratory procedures involving opportunistic pathogenic bacteria, including *Klebsiella pneumoniae*, were performed in accordance with institutional biosafety regulations and standard containment practices for handling potentially pathogenic microorganisms. No experimental manipulations or invasive procedures involving live animals were performed during this study, as only environmental water samples were collected from commercial aquaculture ponds.

### Study period and location

This study was conducted from July 2024 to June 2025 at the Kalasin University Excellent Laboratory for Agricultural and Food Product Standard Testing Center, Faculty of Agricultural Technology, Kalasin University, Thailand.

### Study design

A cross-sectional observational study was designed to investigate the occurrence of ARB and clinically important ARGs in freshwater prawn aquaculture ponds. Water samples were collected quarterly during both rainy and dry seasons to account for seasonal variation. Bacterial isolates were recovered using selective culture techniques and subsequently identified by *16S rRNA* gene sequencing. Antimicrobial susceptibility profiles were determined using the agar disk diffusion method, and PCR assays were employed to detect clinically relevant β-lactamase genes. Descriptive and inferential statistical analyses were performed to evaluate resistance patterns and the prevalence of MDR bacteria.

### Study area description

**Figure 1:**
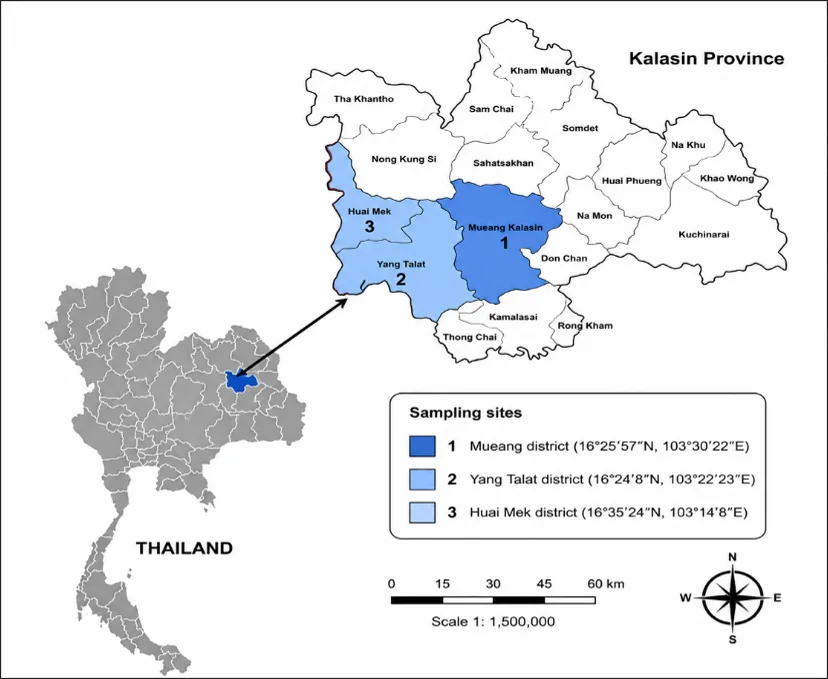
Geographic locations and spatial distribution of giant freshwater prawn (*Macrobrachium rosenbergii*) aquaculture ponds sampled in Mueang, Yang Talat, and Huai Mek districts of Kalasin Province, Northeastern Thailand. Geographic coordinates of the sampling sites are shown to provide geographical and epidemiological context for the study area.

Water samples were collected from earthen aquaculture ponds culturing giant freshwater prawn (*Macrobrachium rosenbergii*) across three districts in Kalasin Province, Northeastern Thailand (Figure 1).

A total of nine earthen aquaculture ponds (three ponds per district) were included in this study. Water sampling was conducted quarterly between July 2024 and June 2025 to capture both rainy and dry seasons. One water sample was collected from each pond at each sampling event, yielding a total of 36 water samples over the study period. Pond selection was based on active cultivation of *M. rosenbergii* and the farmer's willingness to participate in the study.

Organic load was estimated qualitatively based on turbidity and sediment accumulation observed during sampling. No major disease outbreaks were reported during the sampling period, although occasional reductions in water quality and prawn survival were noted by farmers. Ponds were selected using convenience sampling based on accessibility, active freshwater prawn cultivation, and farmers’ willingness to participate in the study.

Basic environmental parameters, including water temperature (28–33°C), pH (7.2–8.1), and dissolved oxygen levels (4.5–6.8 mg/L), were monitored during sampling using portable field meters. Average pond depth ranged from approximately 1.2 to 1.8 m.

### Sample collection and bacterial isolation

Water samples were collected aseptically from earthen aquaculture ponds culturing *M. rosenbergii* across three districts in Kalasin Province, Northeastern Thailand. Samples were collected into sterile 1-L polyethylene bottles and transported on ice to the laboratory for immediate processing within 6 h to preserve microbial viability [12].

To selectively recover environmental ARB associated with local freshwater prawn farming systems, a multi-medium isolation strategy supplemented with 50 µg/mL ampicillin (AMP) was employed. This selective approach was designed to enhance the detection of resistant bacterial populations that may be exposed to antimicrobial selective pressure in earthen aquaculture ponds.

Serial 10-fold dilutions of each water sample were prepared using sterile 0.85% normal saline. A 1-mL aliquot of each dilution (10⁻¹–10⁻²) was plated onto selective media containing 50 µg/mL AMP to isolate potential ARB. AMP was selected as the primary screening antibiotic because β-lactam antibiotics are among the most commonly used antimicrobial agents in freshwater aquaculture systems and are frequently associated with the emergence of MDR environmental bacteria. In addition, AMP supplementation has been widely used to selectively recover Gram-negative ARB, particularly *Aeromonas* spp. and members of the family Enterobacteriaceae, from aquatic environments.

The media used included Luria-Bertani agar (HiMedia Laboratories Pvt. Ltd., Mumbai, India) for general bacterial growth, blood agar base (HiMedia) for detecting hemolytic activity, Streptococcus selection agar (HiMedia), Salmonella-Shigella agar (HiMedia), and thiosulfate-citrate-bile-sucrose agar (HiMedia) for *Vibrio* spp. Following incubation, CFU counts on AMP-supplemented media ranged from approximately 1.2 × 10² to 4.8 × 10⁴ CFU/mL depending on the sampling site and season. Approximately 10–20 colonies displaying distinct morphological characteristics were screened from each sample before selecting representative isolates for further molecular characterization.

Plates were incubated at 37°C for 18–24 h. Colonies exhibiting distinct morphological characteristics were selected and purified through repeated subculturing. Colony morphology, including shape, margin, elevation, and pigmentation, was recorded. Gram staining was performed to classify bacterial groups [13].

Retrospective interviews with pond operators indicated occasional use of oxytetracycline and amoxicillin-based formulations for disease prevention and management. However, complete dosage records and treatment frequencies were inconsistently documented and therefore could not be quantitatively analyzed.

### Molecular identification of bacteria

DNA was extracted from pure bacterial isolates using a standard boiling method. The *16S rRNA* gene region was subsequently amplified using universal primers 27F (5′-AGAGTTTGATCCTGGCTCAG-3′) and 1492R (5′-GGTTACCTTGTTACGACTT-3′), as previously described [14]. PCR reactions were carried out in a total volume of 25 µL using Taq DNA polymerase (Vivantis Technologies Sdn. Bhd., Selangor, Malaysia) and optimized annealing temperatures.

**PCR amplification was performed in a thermal cycler under the following conditions:** initial denaturation at 95°C for 5 min, followed by 35 amplification cycles consisting of denaturation at 95°C for 30 s, annealing at 55–60°C for 30 s depending on primer specificity, and extension at 72°C for 1 min, followed by a final extension at 72°C for 10 min. Positive control strains carrying known resistance genes and nuclease-free water as a negative control were included in each PCR assay.

PCR products were visualized on 1% agarose gels stained with ethidium bromide and subsequently submitted for sequencing using the Macrogen sequencing service (Macrogen Inc., Seoul, South Korea). Resulting sequences were analyzed using the Basic Local Alignment Search Tool against the GenBank database of the National Center for Biotechnology Information (NCBI) using a sequence identity threshold of ≥99% [15]. Phylogenetic trees were constructed using Clustal Omega alignment and the neighbor-joining method [16].

### Antimicrobial susceptibility testing

Antimicrobial susceptibility testing was performed using the agar disk diffusion method on Mueller-Hinton agar according to the guidelines of the Clinical and Laboratory Standards Institute (CLSI) [17]. Inocula were adjusted to the 0.5 McFarland standard and evenly spread on agar plates using sterile cotton swabs.

Commercial antibiotic disks (Oxoid, Thermo Fisher Scientific, Basingstoke, United Kingdom) were used for susceptibility testing and included AMP (10 µg), vancomycin (VAN, 30 µg), azithromycin (AZM, 15 µg), streptomycin (STR, 25 µg), rifampicin (RIF, 5 µg), and chloramphenicol (CHL, 30 µg).

Plates were incubated at 37°C for 24 h, and inhibition zones were measured in millimeters. Results were interpreted as resistant (R ≤15 mm), intermediate (I = 16–20 mm), or susceptible (S ≥21 mm) according to CLSI breakpoints.

All antimicrobial susceptibility tests were performed in triplicate. *Escherichia coli* American Type Culture Collection (ATCC) 25922 and *Staphylococcus aureus* ATCC 25923 were used as quality control strains in accordance with CLSI M100, 31st edition [17].

### Detection of ARGs

Plasmid DNA was extracted from selected antibiotic-resistant isolates using the GF-1 Plasmid DNA Extraction Kit (Vivantis Technologies Sdn. Bhd., Selangor, Malaysia) according to the manufacturer's instructions. Extracted plasmid DNA was quantified using a NanoDrop™ spectrophotometer (Thermo Fisher Scientific, Waltham, MA, USA). Targeted screening for clinically relevant carbapenemase and β-lactamase genes in environmental isolates was conducted to assess the potential dissemination of AMR determinants within freshwater aquaculture ecosystems within a One Health framework.

PCR assays were conducted to detect six resistance genes associated with β-lactamase production, namely *blaTEM, blaSHV, blaOXA, blaKPC-2, blaNDM-1,* and *blaIMP*, using specific primers and previously published protocols [18, 19]. PCR products were resolved on 1.5% agarose gels, stained with ethidium bromide, and visualized under ultraviolet illumination.

The present study focused primarily on plasmid-associated resistance determinants. Chromosomal DNA-associated resistance genes were not specifically investigated.

### Statistical analysis

Descriptive statistics were used to summarize bacterial prevalence and AMR profiles. Differences in resistance frequencies among bacterial species and sampling districts were evaluated using Fisher's exact test or chi-square analysis where appropriate. Statistical significance was defined as p < 0.05. The 95% confidence interval (CI) for MDR prevalence was calculated using the Wilson method.

## RESULTS

### Bacterial identification and phylogenetic analysis

A total of 20 ARB isolates were successfully recovered from water samples collected from three giant freshwater prawn (*M. rosenbergii*) aquaculture ponds in Kalasin Province, Thailand. Based on *16S rRNA* gene sequencing and sequence alignment using the Basic Local Alignment Search Tool, the isolates were taxonomically classified into eight bacterial species across five genera, with sequence identities ≥99% (Table 1).

**Table 1:** Molecular identification and taxonomic characterization of antibiotic-resistant bacterial isolates recovered from giant freshwater prawn (Macrobrachium rosenbergii) aquaculture ponds in Kalasin Province, Thailand, based on 16S rRNA gene sequencing and sequence similarity analysis.

**Isolate**	**GenBank accession number**	**Species**	**Sequence similarity (%)**
YAmp1	PQ432911	*Bacillus cereus*	99
YAmp2	PQ432912	*B. cereus*	99
YBA1	PQ432913	*B. cereus*	100
YBA2	PQ432914	*Bacillus * *wiedmannii*	99
HAmp1	PQ432915	*Klebsiella pneumoniae*	99
HAmp2	PQ432916	*B. * *wiedmannii*	99
HAE1	PQ432917	*Aeromonas * *sanarellii*	99
HAE2	PQ432918	*K. pneumoniae*	99
HTCBS1	PQ432919	*Aeromonas * *veronii*	99
HBA1	PQ432920	*A. * *veronii*	100
HBA2	PQ432921	*A. * *veronii*	99
HSS1	PQ432922	*K. pneumoniae*	99
MAmp2	PQ432923	*A. * *veronii*	99
MAmp3	PQ432924	*A. * *veronii*	100
MAE1	PQ432925	*Enterobacter aerogenes*	99
MAE2	PQ432926	*Aeromonas * *jandaei*	99
MAE3	PQ432927	*Bacillus * *wiedmannii*	99
MSS1	PQ432928	*A. * *veronii*	99
MSS2	PQ432929	*A. * *veronii*	100
MBA2	PQ432930	*Aeromonas * *dhakensis*	99

Identification of bacterial isolates was performed using partial *16S rRNA* gene sequencing followed by sequence similarity analysis against the GenBank database of the NCBI using the Basic Local Alignment Search Tool for nucleotide sequences. Sequence similarity values are presented as percentages relative to the closest reference strains. GenBank accession numbers corresponding to representative isolates generated in this study are provided.

Among the isolates, *Aeromonas veronii* was the predominant species (n = 6), followed by *Bacillus cereus* (n = 3), *Klebsiella pneumoniae* (n = 3), and *Bacillus wiedmannii* (n = 3). Notably, all identified species comprised environmental or opportunistic human pathogens, suggesting anthropogenic influences on pond microbiota through contaminated feed, water sources, or cross-contamination via equipment and surface runoff [10, 20].

**Figure 2:**
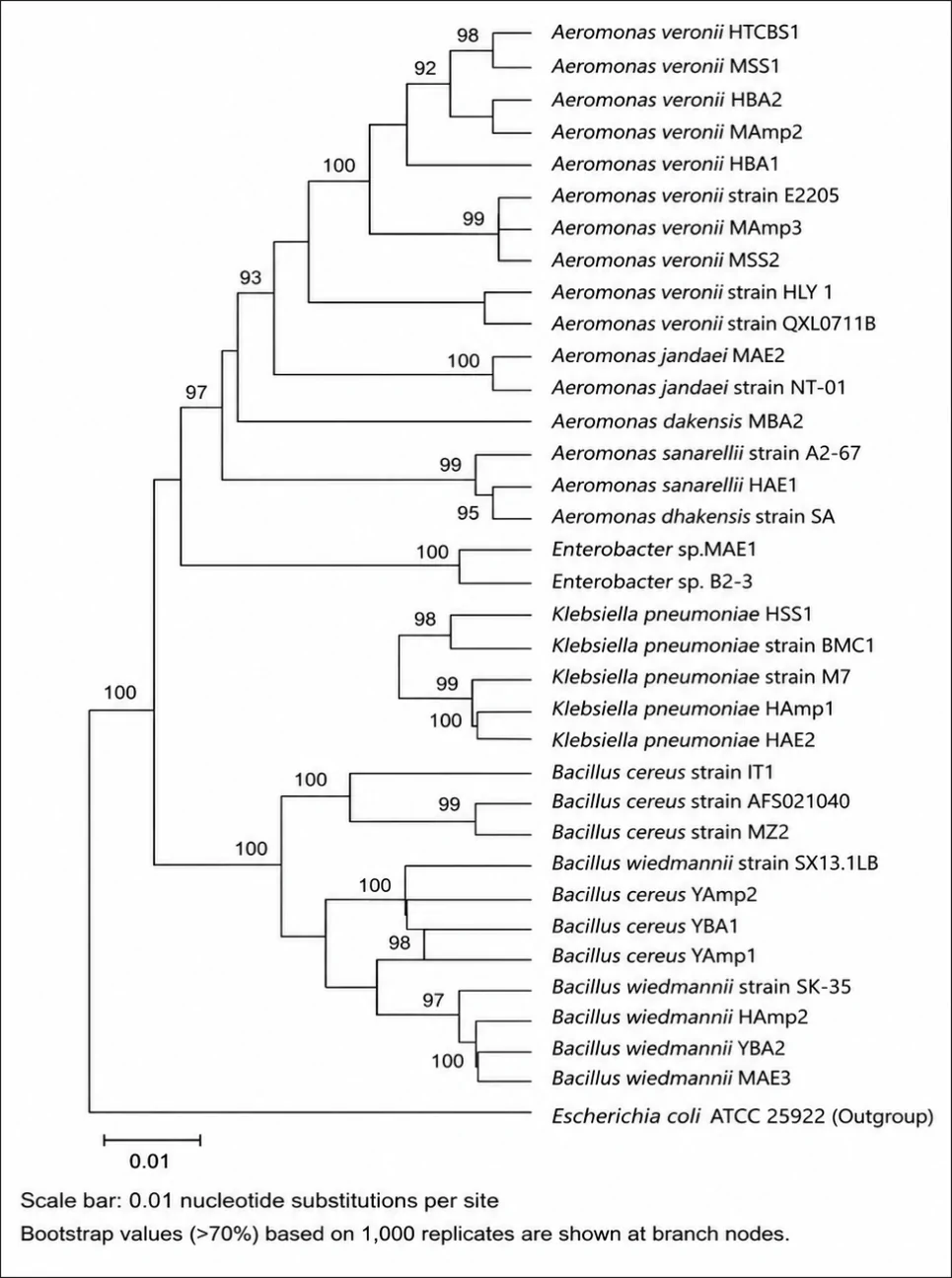
Neighbor-joining phylogenetic tree based on partial *16S rRNA* gene sequences of bacterial isolates recovered from giant freshwater prawn (*Macrobrachium rosenbergii*) aquaculture ponds in Kalasin Province, Thailand. Bootstrap values (>70%) derived from 1,000 replicates are shown at the branch nodes. The scale bar indicates nucleotide substitutions per site. *Escherichia coli* ATCC 25922 served as the outgroup.

The phylogenetic tree constructed from *16S rRNA* gene sequences (Figure 2) showed clear genus-level clustering, reflecting both the taxonomic consistency and environmental diversity of the isolates. Notably, *Aeromonas* spp. formed a well-supported clade, suggesting their ecological dominance under aquaculture conditions. This finding is consistent with previous reports showing a high prevalence of *Aeromonas* species in semi-intensive aquaculture systems characterized by nutrient enrichment and exposure to antimicrobial residues [21].

The occurrence of both environmental species, such as *B. wiedmannii*, and zoonotic species, such as *K. pneumoniae*, highlights the complexity of microbial communities in aquaculture ecosystems and underscores the importance of molecular surveillance to monitor potential public health threats.

### Antimicrobial susceptibility patterns

Antimicrobial susceptibility testing using the agar disk diffusion method demonstrated widespread resistance to multiple antimicrobial classes among the isolates. Fourteen of the 20 isolates (70.0%; 95% CI: 45.7–88.1%) exhibited multidrug resistance (MDR) according to CLSI criteria [17].

**Table 2:** Antimicrobial susceptibility profiles of antibiotic-resistant bacterial isolates recovered from giant freshwater prawn (Macrobrachium rosenbergii) aquaculture ponds in Kalasin Province, Thailand.

**Bacterial species and isolate**	**AMP**	**VAN**	**AZM**	**STR**	**RIF**	**CHL**
*Bacillus cereus* YAmp1	R	R	R	I	R	I
*B. cereus* YAmp2	R	I	I	S	R	S
*B. cereus* YBA1	R	R	I	I	R	S
*Bacillus wiedmannii* YBA2	R	R	S	I	R	R
*Klebsiella pneumoniae* HAmp1	R	R	I	S	R	S
*B. wiedmannii* HAmp2	R	R	I	I	R	R
*Aeromonas sanarellii* HAE1	R	R	S	I	S	S
*Klebsiella pneumoniae* HAE2	R	R	R	S	R	S
*Aeromonas veronii* HTCBS1	R	R	S	R	R	S
*A. veronii* HBA1	R	R	S	R	R	S
*A. veronii* HBA2	R	R	S	R	R	S
*K. pneumoniae* HSS1	R	R	R	S	R	I
*A. veronii* MAmp2	S	S	S	R	R	R
*A. veronii* MAmp3	R	R	I	R	R	R
*Enterobacter aerogenes* MAE1	S	R	R	R	S	S
*Aeromonas jandaei* MAE2	R	R	S	R	R	R
*B. wiedmannii* MAE3	R	R	I	S	R	R
*A. veronii* MSS1	R	R	R	R	R	R
*A. veronii* MSS2	S	R	I	I	R	R
*Aeromonas dhakensis* MBA2	R	R	S	R	S	S

Antimicrobial susceptibility testing was performed using the agar disk diffusion method in accordance with the Clinical and Laboratory Standards Institute guidelines. Inhibition zone diameters were interpreted as resistant (R), intermediate (I), or susceptible (S) according to the breakpoints, where applicable. Multidrug resistance was defined as resistance to at least three antimicrobial classes. AMP = Ampicillin; VAN = Vancomycin; AZM = Azithromycin; STR = Streptomycin; RIF = Rifampicin; and CHL = Chloramphenicol. All antimicrobial susceptibility tests were performed in triplicate.

*A. veronii* exhibited the highest resistance burden, with all six isolates showing resistance to AMP, VAN, RIF, STR, and CHL, together with partial resistance to AZM. Similar MDR profiles were observed in *B. cereus, B. wiedmannii,* and *K. pneumoniae* (Table 2).

The observed resistance pattern likely reflects selective pressure arising from routine antibiotic use in aquaculture systems, either for prophylactic purposes or as growth promoters, particularly in open-pond systems where regulatory control is limited [22]. Intermediate susceptibility (16–20 mm inhibition zones) was also observed in several isolates, suggesting ongoing resistance evolution that may involve plasmid-mediated mechanisms or efflux systems [8].

The emergence of antibiotic resistance in *B. cereus*, traditionally regarded as a low-risk environmental species, further supports the hypothesis that environmental bacteria can acquire clinically important resistance determinants under prolonged antibiotic exposure [23].

No statistically significant differences in MDR prevalence were observed among the three sampling districts (p > 0.05). However, *Aeromonas* spp. exhibited significantly higher frequencies of resistance to RIF and STR than non-*Aeromonas* isolates (p < 0.05).

### Detection of β-lactamase resistance genes

PCR-based molecular screening of plasmid DNA from selected MDR isolates revealed the presence of clinically important β-lactamase genes, particularly *blaSHV* and *blaKPC-2* (Table 3).

Detection of ARGs was performed using PCR assays targeting clinically relevant β-lactamase genes, including *blaTEM, blaSHV, blaOXA, blaKPC-2, blaNDM-1,* and *blaIMP*. Positive amplification was determined by the expected amplicon size on agarose gel electrophoresis. The co-occurrence of *blaSHV* and *blaKPC-2* in *A. veronii* isolate MSS1 may indicate the presence of mobile resistance determinants that contribute to the environmental dissemination of AMR. Positive and negative amplification results are represented by “+” and “−”, respectively.

These genes were detected in multiple isolates of *A. veronii, B. wiedmannii,* and *K. pneumoniae*. The detection of *blaKPC-2*, a carbapenemase gene commonly associated with nosocomial pathogens, is of particular concern because it suggests the potential transfer of resistance determinants from clinical settings to environmental ecosystems [7, 19].

**Table 3:** Distribution and co-occurrence of clinically relevant β-lactamase resistance genes among MDR bacterial isolates recovered from giant freshwater prawn (Macrobrachium rosenbergii) aquaculture ponds in Kalasin Province, Thailand.

**Bacterial species and isolate**	***blaTEM***	***blaSHV***	***blaOXA***	***blaKPC-2***	***blaNDM-1***	***blaIMP***
*Bacillus cereus* YAmp1	−	−	−	−	−	−
*B. cereus* YAmp2	−	−	−	−	−	−
*B. cereus* YBA1	−	−	−	−	−	−
*Bacillus wiedmannii* YBA2	−	+	−	−	−	−
*Klebsiella pneumoniae* HAmp1	−	−	−	−	−	−
*B. wiedmannii* HAmp2	−	+	−	−	−	−
*Aeromonas sanarellii* HAE1	−	−	−	−	−	−
*K. pneumoniae* HAE2	−	−	−	−	−	−
*Aeromonas veronii* HTCBS1	−	−	−	−	−	−
*A. veronii* HBA1	−	+	−	−	−	−
*A. veronii* HBA2	−	−	−	+	−	−
*K. pneumoniae* HSS1	−	+	−	−	−	−
*A. veronii* MAmp2	−	−	−	+	−	−
*A. veronii* MAmp3	−	−	−	+	−	−
*Enterobacter aerogenes* MAE1	−	−	−	+	−	−
*Aeromonas jandaei* MAE2	−	−	−	−	−	−
*B. wiedmannii* MAE3	−	−	−	−	−	−
*A. veronii* MSS1	−	+	−	+	−	−
*A. veronii* MSS2	−	−	−	−	−	−
*Aeromonas dhakensis* MBA2	−	−	−	−	−	−

Most notably, *A. veronii* isolate MSS1 co-harbored both *blaSHV* and *blaKPC-2*, suggesting clustering of MDR determinants. This co-occurrence may indicate the presence of mobile resistance elements and suggests possible dissemination of resistance determinants within aquaculture environments [24]. The environmental occurrence of these genes outside clinical settings further supports the concept that aquaculture systems may function as reservoirs or transmission pathways for antibiotic resistance determinants to human-associated bacterial populations [9].

These findings highlight the importance of incorporating molecular diagnostic approaches into aquaculture AMR surveillance programs and underscore the need for sustainable antibiotic use policies within a One Health framework [11].

Notably, *blaKPC-2* was detected in several environmental isolates, including *A. veronii, Enterobacter aerogenes,* and *K. pneumoniae*, indicating the occurrence of clinically important carbapenemase-associated resistance determinants in freshwater prawn aquaculture environments. Among these isolates, *A. veronii* isolate MSS1 simultaneously harbored *blaSHV* and *blaKPC-2*, suggesting possible clustering of resistance determinants within mobile genetic elements.

To the best of our knowledge, this study represents the first report describing *blaKPC-2*-positive bacteria isolated from *M. rosenbergii* aquaculture ponds in Thailand. The *16S rRNA* gene sequences generated in this study were deposited in the GenBank database under accession numbers PQ432911–PQ432930.

## DISCUSSION

### Occurrence of *blaKPC-2*-carrying bacteria in freshwater prawn aquaculture

To the best of our knowledge, this study is the first report describing *blaKPC-2*-carrying bacteria isolated from *M. rosenbergii* aquaculture ponds in Thailand. The detection of *blaKPC-2* in environmental isolates, particularly in *A. veronii*, is of substantial concern because carbapenemase-producing bacteria have historically been associated mainly with hospital-acquired infections. Although reports of carbapenem-resistant *Aeromonas* spp. in aquatic environments are emerging globally, studies focusing specifically on freshwater prawn aquaculture systems remain limited.

The present findings address an important regional knowledge gap regarding AMR in freshwater aquaculture systems in Northeastern Thailand, where previous AMR investigations have focused predominantly on marine shrimp production systems or fish-associated environments, including studies conducted in Lam Pao Dam [3]. In contrast, the present study specifically investigated earthen pond systems used for *M. rosenbergii* farming in Kalasin Province and demonstrated the predominance of *Aeromonas* spp., consistent with previous reports from aquaculture-impacted environments. However, a high prevalence of MDR isolates and the occurrence of clinically important carbapenemase-associated genes, particularly *blaKPC-2*, were also identified in freshwater prawn pond environments. These findings provide important baseline data for future regional AMR surveillance programs and suggest that earthen pond aquaculture systems may represent distinct ecological niches favoring the persistence and dissemination of AMR determinants.

### Microbial diversity and resistance characteristics

This study provides a comprehensive overview of the microbial diversity and antibiotic resistance characteristics of bacteria isolated from *M. rosenbergii* aquaculture ponds in Kalasin Province, Thailand. The findings reveal the co-occurrence of pathogenic, opportunistic, and environmentally persistent bacterial species, many of which exhibit MDR and harbor clinically significant resistance genes. These results reflect a broader global concern regarding the environmental spread of AMR and its implications for aquaculture sustainability and public health.

The high prevalence of MDR isolates observed in this study may reflect the use of empirical or unregulated antibiotics, which is common in small-scale rural aquaculture systems, where veterinary oversight and antimicrobial stewardship measures may be limited compared with industrial aquaculture operations. Open-pond systems may further facilitate the environmental dissemination of resistant bacteria and resistance genes through water exchange, runoff, and sediment-associated microbial communities.

**Ecological role of** *Aeromonas* **spp.**

The predominance of *A. veronii* among the isolated strains is consistent with its ecological adaptability to aquatic environments, especially those affected by nutrient enrichment and subinhibitory antibiotic concentrations. Previous studies have identified *Aeromonas* spp. as dominant taxa in freshwater ponds due to their metabolic flexibility and tolerance to environmental stressors [1, 21]. This bacterium is also a known pathogen responsible for motile aeromonad septicemia in aquaculture species, contributing to substantial economic losses in Southeast Asia [3]. The consistent detection of *B. cereus*, *B. wiedmannii*, and *K. pneumoniae* across multiple samples suggests that these species may persist in aquaculture systems by associating with organic sediments, detritus, or plankton communities.

### AMR patterns

The observed resistance profiles are alarming. Nearly all isolates were resistant to at least two antibiotics, and many, particularly *A. veronii*, *K. pneumoniae*, and *B. cereus*, were resistant to four or more, including AMP, VAN, RIF, and chloramphenicol. Resistance to VAN and RIF is particularly concerning, as these antibiotics are considered last-resort options in clinical settings [11]. The high levels of resistance observed in *B. cereus*, traditionally considered a low-pathogenicity species, suggest that even commensal or environmental organisms can serve as reservoirs or vectors of resistance genes under aquaculture conditions [8].

### β-lactamase genes and resistance dissemination

Genotypic analysis further supports the phenotypic resistance data. PCR detection of resistance genes revealed the presence of *blaSHV* and *blaKPC-2* in multiple isolates, particularly in *A. veronii* and *K. pneumoniae*. The *blaKPC-2* gene encodes a carbapenemase enzyme that confers resistance to carbapenems, one of the most potent antibiotic classes, and is primarily associated with hospital-acquired infections [7]. Its detection in aquaculture-associated isolates suggests either environmental contamination from anthropogenic sources or the movement of genes facilitated by mobile genetic elements such as plasmids or integrons [19, 24]. Notably, the co-occurrence of *blaSHV* and *blaKPC-2* in *A. veronii* MSS1 reinforces the likelihood of gene clustering, a phenomenon that increases the stability and transmission of resistance determinants across species and environments.

These findings support a growing body of evidence indicating that aquaculture environments, particularly open-pond systems, may serve as important reservoirs and hotspots for the dissemination of ARBs and ARGs [6, 22]. Factors such as poor water quality management, prophylactic antibiotic use, and inadequate treatment regulation may contribute to selective pressures that accelerate AMR development within aquaculture ecosystems. The detection of clinically relevant resistance genes in environmentally adapted species, including *B. wiedmannii*, further suggests that resistance determinants can persist in aquatic microbial communities, sediments, and biofilms, potentially extending beyond harvesting periods and contaminating subsequent production cycles. From a One Health perspective, these findings highlight the interconnected relationships among aquatic environments, animal health, and public health. However, additional investigations involving exposure assessments, food-chain analyses, and gene-transfer studies are necessary to better evaluate the actual public health implications of freshwater prawn aquaculture systems [11, 20].

### Detection of *blaSHV* in *B. wiedmannii*

Interestingly, *blaSHV* was also detected in *B. wiedmannii*, an environmental species not commonly associated with clinically important β-lactamase genes. Although *Bacillus* spp. are frequently detected in aquaculture environments and are often considered part of the environmental microbiota, the occurrence of *blaSHV* in these isolates may suggest environmental acquisition of resistance determinants under antibiotic selective pressure. Similar observations have occasionally been reported in environmental bacterial communities, although their ecological significance remains poorly understood.

### Practical and policy implications

This study highlights several critical implications. First, it supports the call for more stringent antibiotic use regulations in aquaculture, including the adoption of antimicrobial stewardship programs and the enforcement of withdrawal periods before harvest. Second, the integration of molecular diagnostics, including quantitative PCR and metagenomics, into routine monitoring programs would enable early detection of resistance trends and guide targeted interventions. Third, alternative disease management approaches, such as probiotics, vaccination, and improved biosecurity, should be prioritized to reduce reliance on antibiotics [2].

From a policy perspective, these findings support the need for improved antimicrobial stewardship and routine AMR surveillance within Thailand’s freshwater aquaculture sector. The integration of aquaculture-associated AMR monitoring into national One Health action plans may contribute to more sustainable disease management strategies and reduce the environmental dissemination of clinically important resistance determinants.

### Limitations of the study

Several limitations should be considered when interpreting the findings of this study. First, bacterial isolation relied on culture-based methods using AMP-supplemented selective media, which may underestimate the diversity of unculturable or non-target bacterial populations present in aquaculture environments. Second, the number of representative isolates included in molecular analyses was relatively limited (n = 20), potentially restricting broader ecological interpretation. Third, detailed farm-level antimicrobial usage records were inconsistently available, limiting direct assessment of associations between antibiotic use practices and resistance profiles. In addition, environmental contamination from external water sources or agricultural runoff could not be completely excluded. The absence of non-aquaculture reference sites or low-antibiotic-use control ponds limited comparative ecological interpretation of resistance prevalence in the present study. Although the co-occurrence of *blaSHV* and *blaKPC-2* may indicate the presence of mobile resistance determinants, no plasmid sequencing, conjugation assays, or whole-genome sequencing was conducted to confirm gene-transfer or mobility. Future investigations integrating metagenomics, quantitative PCR, and resistome analysis are therefore recommended.

## CONCLUSION

This study demonstrated the occurrence of ARB harboring clinically important β-lactamase resistance genes in giant freshwater prawn aquaculture ponds in Kalasin Province, Thailand. A total of 20 bacterial isolates representing eight species and five genera were identified, with *A. veronii* being the predominant species. Fourteen isolates (70.0%; 95% CI: 45.7–88.1%) exhibited MDR phenotypes, and molecular analyses revealed the presence of clinically relevant resistance genes, particularly *blaSHV* and *blaKPC-2*. Notably, the co-occurrence of *blaSHV* and *blaKPC-2* in *A. veronii* isolate MSS1 and the detection of *blaKPC-2*-positive isolates in multiple bacterial species represent important findings and provide the first evidence of *blaKPC-2*-carrying bacteria associated with freshwater prawn aquaculture ponds in Thailand.

These findings highlight the potential role of freshwater aquaculture systems as environmental reservoirs and dissemination pathways for AMR determinants. The results emphasize the importance of implementing prudent antimicrobial use practices, strengthening biosecurity measures, and incorporating routine molecular surveillance into disease management programs. Furthermore, the integration of aquaculture-associated AMR monitoring within a One Health framework may contribute to more sustainable aquaculture production and help mitigate the spread of clinically important resistance determinants.

A major strength of this study was the integrated approach that combined culture-based isolation, molecular identification, antimicrobial susceptibility profiling, and targeted detection of β-lactamase genes, thereby generating valuable baseline data for freshwater aquaculture systems in Northeastern Thailand.

Future studies should incorporate metagenomics, quantitative PCR, whole-genome sequencing, and resistome analyses to better elucidate the ecology, transmission dynamics, and persistence of ARGs in aquaculture ecosystems. Additional investigations involving environmental exposure pathways and food-chain transmission are also warranted to clarify the public health significance of resistant bacteria associated with freshwater aquaculture.

In conclusion, the present findings provide important baseline information for AMR surveillance in freshwater prawn farming and underscore the need for integrated antimicrobial stewardship and sustainable disease management strategies. Strengthening surveillance and adopting preventive approaches will be essential for safeguarding aquaculture productivity, environmental health, and public health under the One Health paradigm.

## DATA AVAILABILITY

The *16S rRNA* gene sequences generated during this study were deposited in the GenBank database under accession numbers PQ432911–PQ432930. Supplementary antimicrobial susceptibility data, environmental metadata, and raw inhibition zone measurements are publicly available in the Figshare repository (https://doi.org/10.6084/m9.figshare.32298075).

## GENERATIVE AI DECLARATION

The authors declare that generative artificial intelligence (AI) tools were used solely to improve language, grammar, and readability during manuscript preparation. All scientific content, data analysis, interpretation of results, and conclusions were developed and verified by the authors. The authors take full responsibility for the accuracy, integrity, and originality of the work presented, and no AI tool was listed as an author.

## AUTHORS’ CONTRIBUTIONS

KP, PK, TB, AT, UK, and NK: Conceived and designed the study, sample collection, bacterial isolation, molecular identification, antimicrobial susceptibility testing, detection of ARGs, and statistical analyses. KP, AT, and NK: Interpreted the data and results. KP and NK: Drafted and critically revised the manuscript. All authors have read and approved the final version of the manuscript.
